# Hollow CoS/C Structures for High-Performance Li, Na, K Ion Batteries

**DOI:** 10.3389/fchem.2022.845742

**Published:** 2022-03-10

**Authors:** Yan Liu, Xiangkun Li, Fengling Zhang, Leqing Zhang, Tao Zhang, Changshuan Li, Zhicheng Jin, Yueying Wu, Zhongyu Du, Huiwen Jiao, Ying Jiang, Yuliang Yan, Qiang Li, Weijin Kong

**Affiliations:** ^1^ College of Physics, University-Industry Joint Center for Ocean Observation and Broadband Communication, Qingdao, China; ^2^ Weihai Innovation Institute, Qingdao University, Weihai, China

**Keywords:** CoS, porous carbon, Li ion batteries, Na ion batteries, K ion batteries

## Abstract

Alkali ion (Li, Na, and K) batteries as a new generation of energy storage devices are widely applied in portable electronic devices and large-scale energy storage equipment. The recent focus has been devoted to develop universal anodes for these alkali ion batteries with superior performance. Transition metal sulfides can accommodate alkaline ions with large radius to travel freely between layers due to its large interlayer spacing. Moreover, the composite with carbon material can further improve electrical conductivity of transition metal sulfides and reduce the electron transfer resistance, which is beneficial for the transport of alkali ions. Herein, we designed zeolitic imidazolate framework (ZIF)–derived hollow structures CoS/C for excellent alkali ion (Li, Na, and K) battery anodes. The porous carbon framework can improve the conductivity and effectively buffer the stress-induced structural damage. The ZIF-derived CoS/C anodes maintain a reversible capacity of 648.9, and 373.2, 224.8 mAh g^−1^ for Li, Na, and K ion batteries after 100 cycles, respectively. Its outstanding electrochemical performance is considered as a universal anode material for Li, Na, and K ion batteries.

## Introduction

Energy storage devices such as rechargeable batteries are the cornerstone of sustainable energy. With the rapid demand for large industrial devices, such as aerospace, national grid, electric vehicles, and so on, novel rechargeable batteries with high density and long cycle need to meet the supply comprehensively (Tarascon and Armand, 2001; Armand and Tarascon, 2008; Huang et al., 2020; Hongsen Li et al., 2021; Qiang Li et al., 2021; Zhang et al., 2021). Among them, lithium ion batteries (LIBs) are used as the source of hybrid electric vehicles at the earliest stage (Hosaka et al., 2020). The long-term use of lithium has made it expensive and scarce in the earth’s crust (Vaalma et al., 2018). Therefore, sodium ion batteries (SIBs) and potassium ion batteries (PIBs) have gradually developed into high-quality alternatives to novel rechargeable batteries (Geng et al., 2018; Loaiza et al., 2019; Zhaohui Li et al., 2021). Sodium and potassium ions are abundant and inexpensive, and the lower standard electrode potentials of Na/Na^+^ and K/K^+^ can reduce the cutoff potential of the available negative electrode in the absence of metal sodium or potassium deposition (Mao et al., 2018; Liu et al., 2021).

However, the commercial graphite anode cannot ensure high density because of its low theoretical capacity, which cannot meet the application of LIBs in large-scale energy storage devices. Moreover, the large radius and heavier weight of sodium ions and potassium ions also make it impossible to shuttle freely and reversibly in the graphite lattice (Xu et al., 2017; Mao et al., 2018; Hongkang Wang et al., 2020). Therefore, the current exploration of potential anode materials with superior reversible capacity and excellent cycle/rate performances is an urgent need for rechargeable batteries including LIBs, SIBs and PIBs. Transition metal sulfides have attracted numerous attentions as anode materials in electrochemical energy storage due to the high specific capacities (Peng et al., 2016; Geng et al., 2018). Among them, cobalt sulfide has been widely studied in catalysis, capacitors, and LIBs by virtue of its unique physical, chemical, and electronic properties (Xie et al., 2013; Peng et al., 2014; Chen et al., 2016; Xu et al., 2018; Song and Yao, 2020), but the application of this electrode material to the negative electrode of SIBs and PIBs is rarely reported. In order to meet the large-scale application of high-performance alkaline ion batteries, it is necessary to design the structure of cobalt sulfide to avoid the structural damage in the cycle process. As an excellent precursor for constructing nanostructures and hollow structures, zeolitic imidazolate framework (ZIF-67) has adjustable physical and chemical properties and large specific surface area (Wang et al., 2016; Zhao et al., 2017; Lu et al., 2020; Yi et al., 2020). ZIF-derived cobalt sulfide hollow structure can effectively alleviate the structural damage caused by stress. The carbon layer framework greatly improves the conductivity of the electrode, and this structure has a huge cavity and porous carbon wall, which makes electrolyte and ions easy to enter, reduces the electron transfer resistance, and shortens the ion diffusion path (Xia et al., 2015; Liu et al., 2017).

Combined with the advantages of the above structural design, we designed a hollow ZIF-derived cobalt sulfide anode material. CoS was uniformly dispersed in the carbon layer of the ZIF framework in the form of nanocrystals. This kind of anode material has excellent electrochemical performance in LIBs, SIBs, and PIBs. The long-term cycling stability and rate capability are benefited from the integrated favorable structural characteristics of electrode. The carbon framework ensures the full penetration of electrolyte and shortens the diffusion path of ions. The porous hollow structure can also be well maintained upon the insertion/extraction of ions with large radius, which lays an effective foundation for the design of anode materials for the next generation of high-performance rechargeable batteries.

## Experimental Details

### Synthesis Procedure of ZIF-67 Template

In a typical process, 0.873 g of cobalt nitrate hexahydrate (Co(NO_3_)_2_·6H_2_O) was first dissolved in 30 mL methanol to form homogeneous solution. And another transparent solution can be obtained through dissolving 0.984 g of 2-methylimidazole into 10 mL of methanol. Afterward, these two solutions were mixed and shaken vigorously for 3 min, and the resulted mixed solutions were aged for 12 h at room temperature. After precipitation, centrifugation, and carefully washing with methanol, the corresponding precipitates were finally collected and dried overnight in an oven at 80°C.

### Preparation of Hollow CoS/C Nanoparticles

In order to obtain hollow CoS/C nanoparticles, 25 mg ZIF-67 powder was first dispersed in 6 mL of ethanol with ultrasonication for 30 min to form a homogenous suspension. Then, 0.1875 mM of thioacetamide (dissolved in 2 mL ethanol) was added into the suspension and stirring in an oil bath for 1 h at 90°C. After that, the suspension was cooled down to room temperature and purified with deionized (DI) water and ethanol for several purification cycles. Finally, black products can be obtained under 50°C drying condition.

### Preparation of CoS Nanoparticles

The pure CoS was prepared to compare with CoS/C; 190.4 mg of cobaltous chloride hexahydrate (CoCl_2_⋅6H_2_O) and 134.4 mg of sulfourea (CS(NH_2_)_2_) were dissolved in 40 mL of ethylene glycol. The obtained solution was then transformed into a Teflon-lined autoclave and kept at 180°C for 12 h. The precipitation was collected by centrifugation and washed with water and then dried in the vacuum oven at 70°C for 12 h.

### Material Characterizations

The crystal structure was determined through X-ray diffraction (XRD) measurement with a high-intensity Cu Kα radiation (*λ* = 1.5406 Å). Scanning electron microscopy (SEM) images were used to observe the surface morphology through JSM-6700F microscope. High-resolution transmission electron microscopy (HR-TEM) and selected area electron diffraction (SAED) results were obtained through JEOL 100 CX microscope. X-ray photoelectron spectroscopy (XPS; Thermo ESCALAB 250XI) was used to analyze the chemical bonding of hollow CoS/C nanoparticles, and the corresponding spectrum was collected by a Kratos-Axis spectrometer under monochromatic Al Kα (1,486.6 eV) x-ray radiation (15 kV, 10 mA) with an energy resolution of 0.45 eV/(Ag 3d_5/2_) and C 1 s (284.5 eV) band correction. Thermogravimetric analysis (TGA; Pyris Diamond6000 TG/DTA; PerkinElmer Co., USA) can analyze the thermal properties of samples in air over a temperature range of 20°C–700°C with a heating rate of 10°C min^−1^. N_2_ adsorption/desorption isotherms were performed on a Micromeritics ASAP 2020 instrument to test the Brunauer–Emmett–Teller (BET) surface areas and porosity for as-prepared sample. The Raman test was performed on a LabRAM HR Evolution spectrograph (form France; Horiba Scientific, Longjumeau, France) with a laser at *λ* = 532 nm (He/Ne laser, <10 mW) at the range of 600–1,800 cm^−1^.

### Electrochemical Measurements

The electrochemical performance of the CoS/C nanoparticles was measured by assembled LIBs. The electrode can be obtained by homogenously mixing the active material, super-p, and sodium carboxymethyl-cellulose in DI with a mass ratio of 7:2:1; the prepared slurry was uniformly coated onto a copper foil and then dried under vacuum at 60°C for 12 h. For half-cell, lithium metal was used as the counter and reference electrode; the electrolyte was 1 M LiPF_6_ in 1:1 vol/vol mixture of ethylene carbonate and diethyl carbonate. Celgard 2,250 film was utilized as the separator (Whatman). All electrochemical measurements were carried out at room temperature. Cyclic voltammetry (CV) and electrochemical impedance spectroscopy were performed on a CHI660E electrochemical workstation. Galvanostatic charge–discharge measurements were performed under a LAND-CT2001A instrument.

## Results and Discussion

**FIGURE 1:**
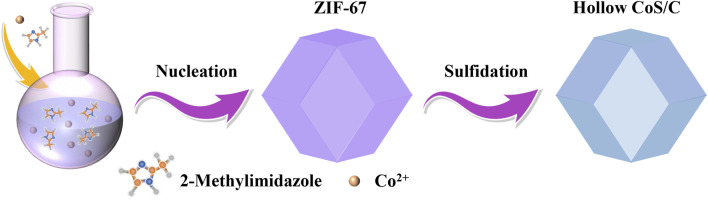
Illustrations of the fabrication processes of ZIF-derived hollow CoS/C sample.

**FIGURE 2:**
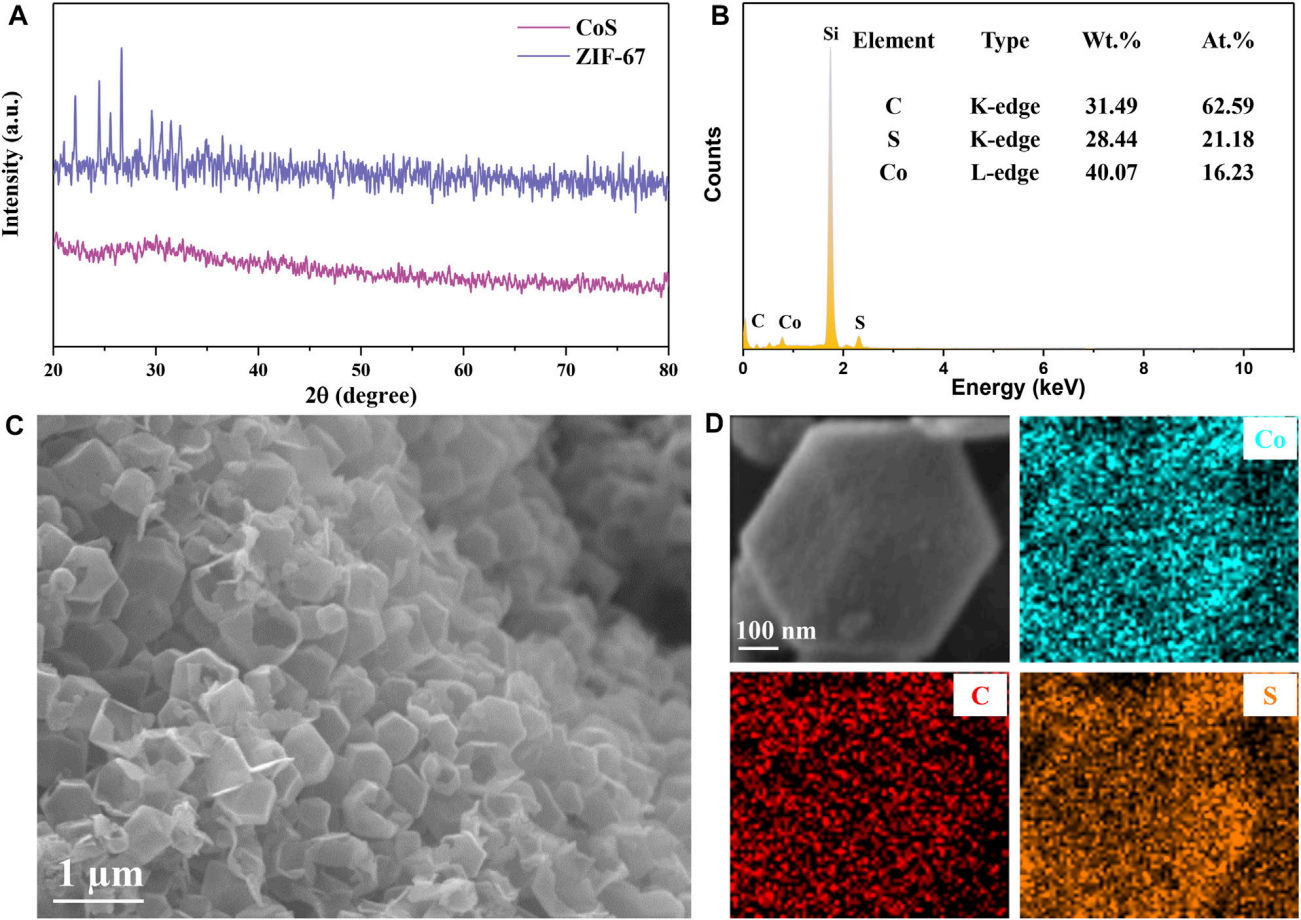
**(A)** X-ray patterns of as-prepared ZIF-67 precursor and ZIF-derived hollow CoS/C. **(B)** EDS spectrum of ZIF-derived CoS/C. The Si element comes from the Si substrate used for SEM characterizations. **(C)** SEM images and **(D)** EDS mapping of ZIF-derived CoS/C, confirming the presence of Co, C, and S without any impurities.

After natural nucleation of the mixed homogeneous solution, the formed ZIF-67 precursor shows a uniform nanocrystalline polyhedron structure. Hollow CoS/C nanocomposites with the same polyhedron structure can be obtained accompanied with the sulfidation process (Figure 1). The powder XRD results are shown in Figure 2A; it can be seen that there are obvious diffraction peaks in the diffraction patterns of ZIF-67 precursor (Zhou et al., 2017). By contrast, no obvious peaks can be observed in diffraction curves of CoS/C obtained by precursor after sulfidation, which is consistent with the previous reported results (Hu et al., 2016; Xu et al., 2018). This kind of phenomenon can be attributed to the amorphous nature of CoS/C materials; in other words, CoS/C nanoparticles exhibit small crystalline grains and poor crystallinity. And then, the chemical composition of CoS/C was analyzed through energy-dispersive X-ray spectroscopy (EDS), and the corresponding results are exhibited in Figure 2B. The EDS results show that the atomic ratio of Co and S is approximately ∼1:1, suggesting the successful preparation of ZIF-derived hollow CoS/C samples. And the Si element comes from the Si substrate used for SEM characterizations. The surface morphology of CoS/C was characterized through SEM measurement; as can be seen from Figure 2C, the CoS/C samples show well-defined polyhedron structure with hollow core. In addition, the existence of Co, C, and S elements can be observed in the elemental mappings of as-prepared CoS/C sample shown in Figure 2D, demonstrating that all these elements are uniformly distributed in the hollow CoS/C sample.

**FIGURE 3:**
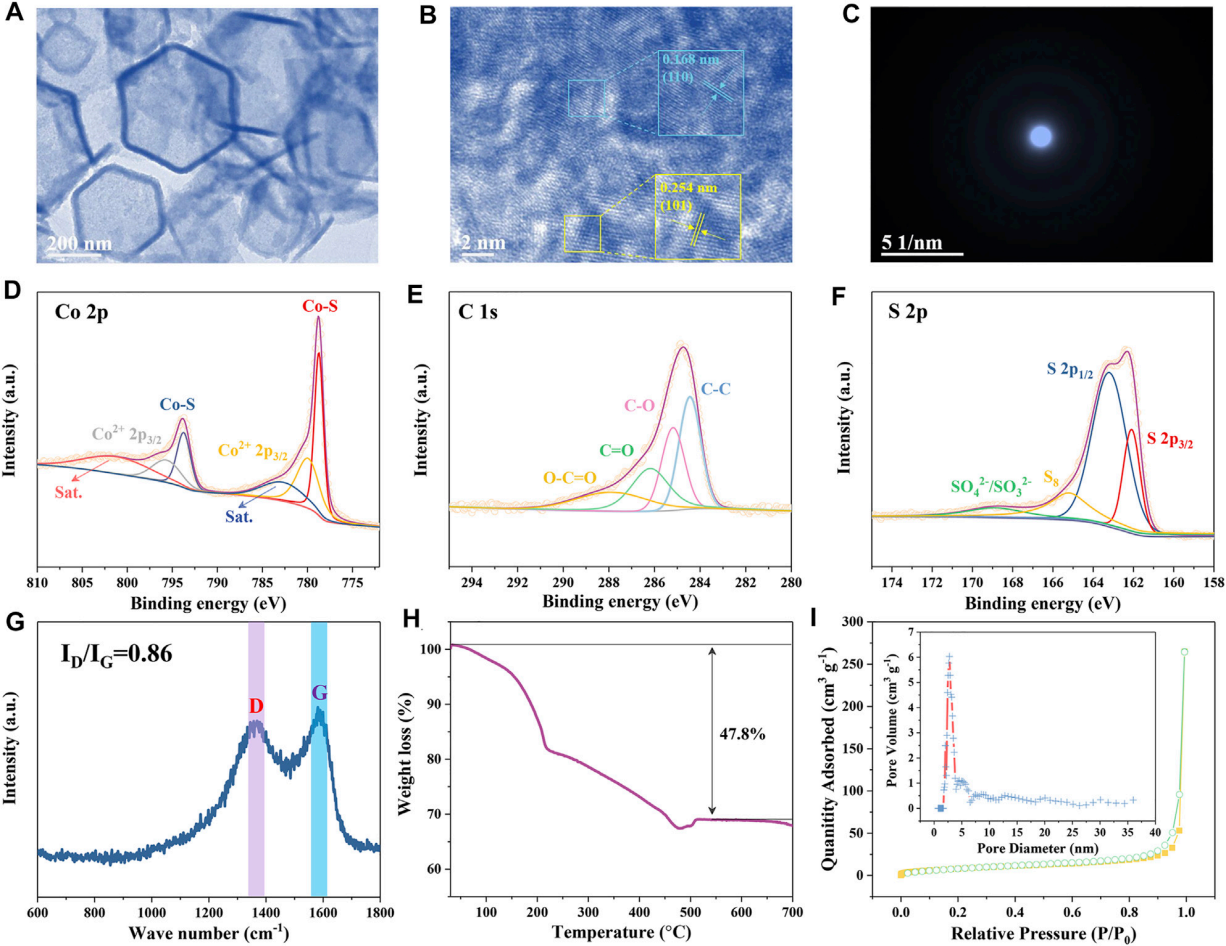
TEM images **(A,B)** and SAED pattern **(C)** of CoS/C. **(D–F)** XPS results of CoS/C. **(G)** Raman spectrum, **(H)** TGA curve, and **(I)** N_2_ absorption/desorption isotherms of CoS/C (inset: corresponding pore size distribution).

The TEM image in Figure 3A further reveals that the ZIF-derived CoS/C sample is a hollow hexagonal structure. Moreover, from the HR-TEM as shown in Figure 3B, the lattice fringes of 0.168 and 0.254 nm can be assigned to the (110) and (101) plane of CoS, respectively (Peng et al., 2016; Pan et al., 2021). The HR-TEM results further confirmed the existence of CoS in the hollow hexagonal framework. Besides, the SAED was performed to characterize the crystallinity of obtained ZIF-derived CoS/C materials. As shown in Figure 3C, no diffraction spots or diffraction rings can be observed in SAED patterns, indicating the obtained CoS/C featured in amorphous nature; this might be caused by the small particles and poor crystallinity (Cui et al., 2013).

Furthermore, the chemical composition of CoS/C and chemical valence states of Co, C, and S were analyzed by XPS measurements. Figure 3D presents the Co 2p XPS spectrum of the hollow CoS/C nanocomposite. Two main peaks at 778.7 and 793.7 eV can be assigned to the Co-S bond, whereas the peaks located at 780.0 and 795.7 eV can be attributed to the Co^2+^ 2p_3/2_ and Co^2+^ 2p_1/2_, respectively. In addition, peaks at 782.9 and 801.3 eV can be detected, indicating the existence of satellite peaks. The C 1s XPS spectrum is given in Figure 3E, where carboxylate carbon O–C=O, carbonyl carbon C=O, and C–O locates at 288.1, 286.2, and 285.2 eV, respectively, and the peak at 284.4 eV can be ascribed to C–C bonds. Peaks in XPS spectrum of S 2p shown in Figure 3F are located at approximately 162.1 and 163.2 eV, corresponding to S 2p_3/2_ and S 2p_1/2_, respectively. And the peak with 165.3 eV binging energy is assigned to S–S bond. Moreover, another peak at the 169.0 eV position is attributed to the surface sulfur in the high oxidation state (SO_4_^2-^/SO_3_^2-^) (Yu et al., 2019; Yun-Kai Wang et al., 2020). And then, Raman spectrum measurement was also carried out to investigate the nature of carbon. As shown in Figure 3G, two distinguished carbon bands can be identified from the Raman spectroscopy, which are related to the D and G bands. The D band can be attributed to the sp^2^ hybridization of carbon, whereas the G band is related to the degree of graphitization. The peak intensity ratio of D/G band is 0.85; the disorder of carbon is beneficial to the alkali metal ion storage (Hu et al., 2020; Ruan et al., 2020).

The TGA result of CoS/C is displayed in Figure 3H. The carbon component will be burned out, and the CoS will be converted to Co_3_O_4_ through chemical reaction 3CoS (s) + 2O_2_ (g) = Co_3_O_4_ (s) + 3S (g). The calculated carbon content in ZIF-derived CoS/C is approximately 30 wt%, which is consistent with the above EDS spectrum. The BET-specific surface area of CoS/C was measured by N_2_ adsorption–desorption measurement, and the pore size distribution was determined through quenched solid density functional theory. As can be seen from Figure 3I, the BET surface area is ∼21.60 m^2^ g^−1^ with micropore peaks at 3.5 nm. The porous structure containing a large number of mesopores in ZIF-derived CoS/C is beneficial to the ionic adsorption, buffering the volume expansion during the electrochemical process and supporting high rate performance (Ruan et al., 2020).

**FIGURE 4:**
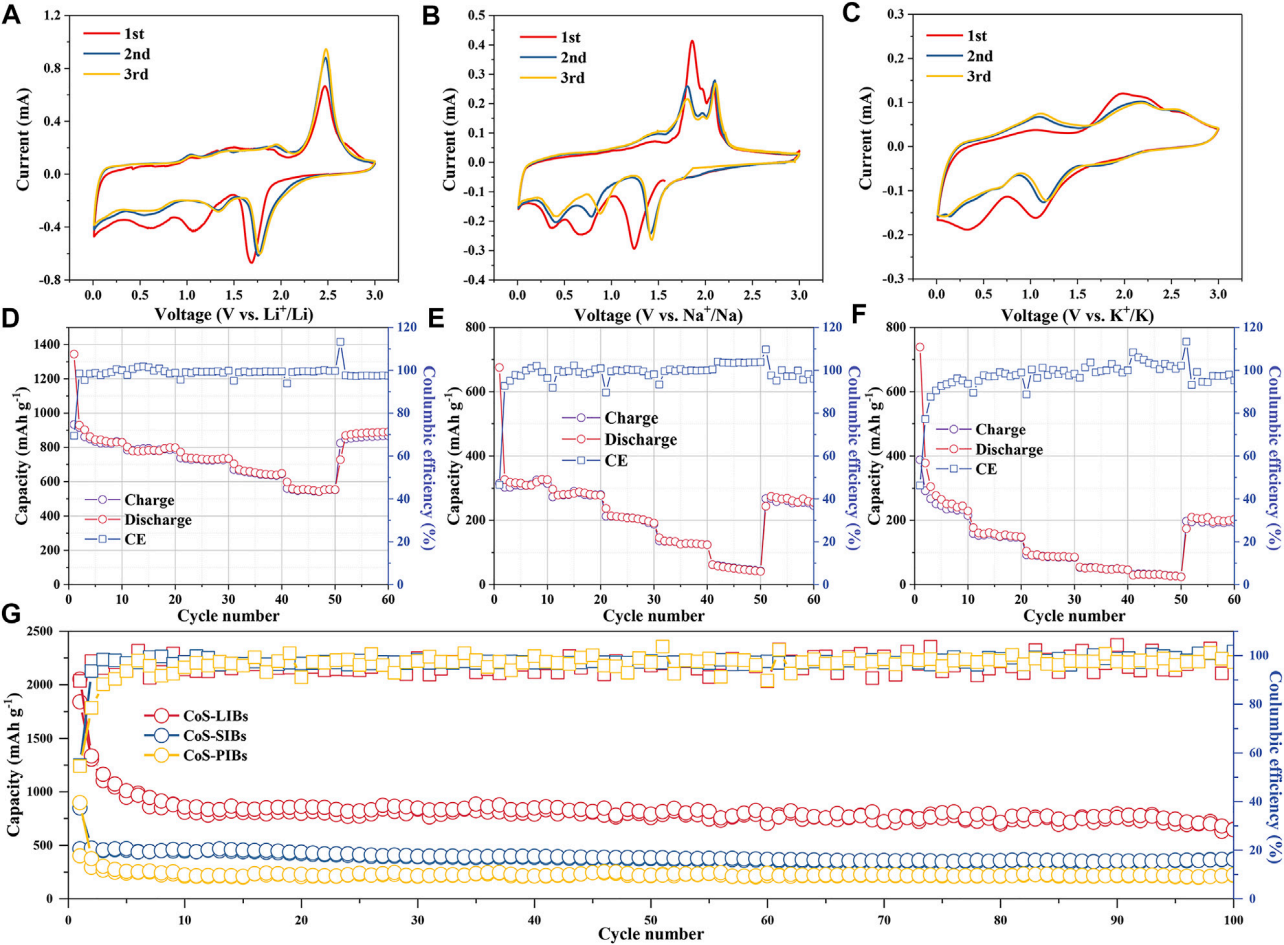
CV measurement of ZIF-derived CoS/C electrode in **(A)** LIBs, **(B)** SIBs, and **(C)** PIBs for the first three cycles at a scan rate of 0.2 mV · s^−1^. Rate performance of CoS/C electrode in **(D)** LIBs, **(E)** SIBs, and **(F)** PIBs. **(G)** Cycling capabilities for CoS/C–LIBs, CoS/C–SIBs, and CoS/C–PIBs at a current density of 0.2 A g^−1^.

The electrochemical performance of CoS/C was investigated in LIBs, SIBs, and PIBs for comparison. The CV measurements of CoS/C electrode in LIBs, SIBs, and PIBs for the first three cycles are presented in Figures 4A–C, respectively. The redox peaks in CV curves of all three batteries can well correspond to the platforms of discharge/charge curves (Supplementary Figure S1). For CoS/C–LIBs, a cathodic peak locates at 1.7 V in the first sweep cycle can be attributed to the initial insertion of lithium to form Li_x_CoS_2_ (Wu et al., 2015), whereas the peaks at approximately 1.1 V in the first cycle and then decreased in the following scans are due to the conversion reaction of CoS to metallic Co and the formation of Li_2_S (Chen et al., 2016). Following the charging process, the oxidation peak at approximately 2.4 V can be attributed to the sulfidation of reduced Co nanoparticles. As for the CoS/C–SIBs, the peak at approximately 1.25 V in the first cathodic scan represents the initial insertion of sodium process, and the peak at 0.7 V is attributed to the formation of SEI film (Chen et al., 2016). For CoS/C–PIBs, the peak related to the initial insertion of potassium locates at 1.1 V and the peak attributed to SEI film formation are located at 0.4 V. For all three kinds of batteries, the peak intensity of the SEI film decreases with the increasing number of cycles, indicating that the side reactions such as electrolyte decomposition weaken gradually, which is consistent with the increase in Coulombic efficiency. Owing to different charge–discharge mode, the changes of Coulombic efficiency reflected by CV peak intensity variations are not as obvious as that under the galvanostatic charge–discharge mode (Ge et al., 2020). Besides, the intensity of redox peaks in LIBs is stronger than that in SIBs and PIBs, indicating that LIBs, SIBs, and PIBs show different reaction degrees based on different alkali metal ions. The storage behaviors of K and Na ions have lower absolute value changes of Gibbs energy, and the kinetics of charging and discharging process in PIBs are more sluggish than those in LIBs and SIBs, which can be ascribed to the larger ionic radius of K^+^ than Na^+^ and Li^+^ (Loaiza et al., 2019).

Figure 4D shows the rate performance of the ZIF-CoS/C electrode in LIBs, which delivers discharge specific capacities of 834.9, 798.4, 734.6, 644.9, and 553.8 mAh g^−1^ at 0.1, 0.2, 0.5, 1 and 2 A g^−1^ current density, respectively. When the current density recovers to 0.1 A g^−1^, the discharge capacity of 890.0 mAh g^−1^ can still be maintained, suggesting a superior rate capability. Moreover, the rate performance of ZIF-CoS/C electrode in SIBs and PIBs are also investigated, and the results are shown in Figures 4E, F, respectively. Compared with LIBs, specific capacity of CoS/C electrode in SIBs and PIBs decays rapidly, which is related to the larger radius of Na^+^/K^+^ than Li^+^, and the result is consistent with the CV curves. Although the capacity is not as high as LIBs, the capacity still recovers well when the current density returns to 0.1 A g^−1^ in SIBs and PIBs. This phenomenon suggests that the unique structure of CoS/C nanoparticles and uniform hollow carbon framework ensure its excellent structural stability, which can well accommodate large radius ions to shuttle inside and buffer volume changes. Furthermore, the comparison of long-term cycling performance of ZIF-derived CoS/C electrode in corresponding ion batteries with the voltage range of 0.01–3 V is presented in Figure 4G. After 100 cycles, CoS/C anode can obtain a discharge capacity of 648.9, 373.2, and 224.8 mAh g^−1^ for LIBs, SIBs, and PIBs, respectively. The lower capacity of PIBs and SIBs compared with that in LIBs can be attributed to large alkali metal ions radius and incomplete conversion reaction of CoS/C nanoparticles. More importantly, after 100 cycles, the capacity remained stable without obvious attenuation. The superior cycling stability of both three kinds of alkali metal ion batteries further proved the robust structure stability of the electrode upon cycling as shown in Supplementary Figure S2. Compared with pure CoS (Supplementary Figure S3), CoS/C exhibits better cycling stability and higher capacity, which proves that the unique ZIF structure is beneficial to the structural stability of the electrode materials, and the introduction of carbon can increase the electrical conductivity, thus improving the capacity.

**FIGURE 5:**
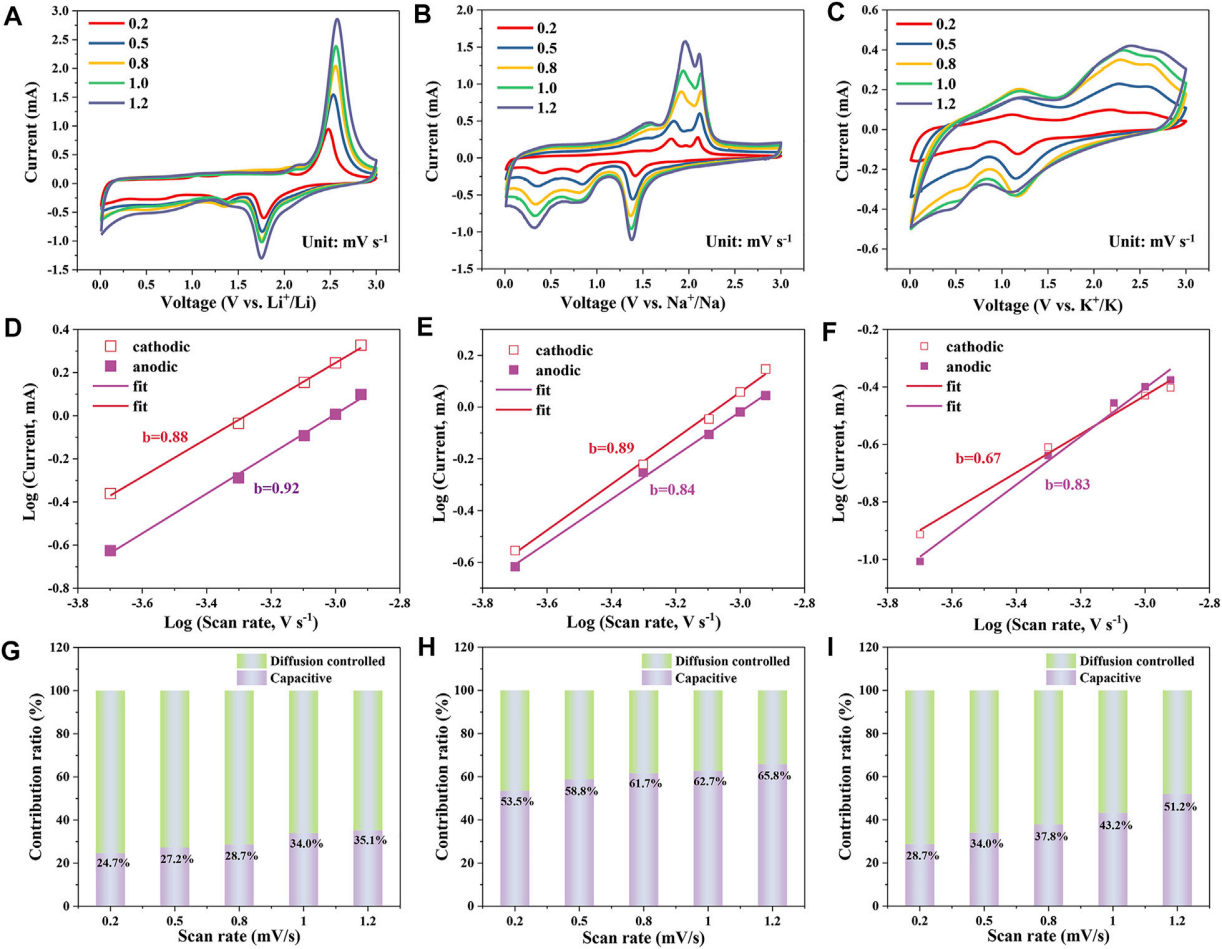
CV measurements of CoS/C electrode in **(A)** LIBs, **(B)** SIBs, and **(C)** PIBs with various sweep rates. Log (current, mA)–log (sweep rate, mV s^−1^) curves of CoS/C electrode in **(D)** LIBs, **(E)** SIBs, and **(F)** PIBs at the typical peaks. Normalized contribution ratio of diffusion-controlled and capacitive capacities for **(G)** CoS/C–LIBs, **(H)** CoS/C–SIBs, and **(I)** CoS/C–PIBs with various scan rates.

In order to investigate the reaction kinetics of the alkali metal ion storage, the CV curves of CoS/C-LIBs, CoS/C-SIBs and CoS/C-PIBs with different sweep rates ranging from 0.2 mV s^−1^–1.2 mV s^−1^ were examined, and the corresponding results were provided in Figures 5A–C, respectively. It can be seen from the results that they displayed similar CV scan shape except that the peaks grow broader with increasing sweep rates. The electrochemical reaction kinetics can be further analyzed by the following equation:

$$i=av^{b}$$ (1)

where *i* is the peak current density, *a* and *b* are adjustable parameters, *v* is the scan rate, *b* = 0.5 implies the diffusion-controlled behavior, and *b* = 1 indicates surface-controlled capacitive behavior. As a consequence, the value of *b* obtained by the relation between log(*v*) and log(*i*) can be used to investigate the reaction kinetic behavior. For CoS/C-LIBs, the fitted *b* values of cathodic and anodic peaks were 0.88 and 0.92 (Figure 5D), respectively, which indicates the electrochemical reaction kinetics of CoS/C-LIBs is diffusion-controlled and surface-controlled capacitive behavior together. In the case of CoS/C-SIBs and CoS/C-PIBs (Figures 5E, F), the fitted *b* values from cathodic/anodic peaks are 0.89, 0.84, and 0.67, 0.83, respectively. These results indicate the electrochemical reaction kinetics of CoS/C-SIBs and CoS/C-PIBs is similar to CoS/C-LIBs, which dominated through diffusion-limited and surface-controlled capacitive behavior together. To further quantify the specific contribution of capacitive capacity and diffusion capacity, we conducted calculations through the following equation:

$$i\left(V\right)=k_{1}v+k_{2}v^{1/2}$$ (2)

$$i\left(V\right)/v^{1/2}=k_{1}v^{1/2}v+k_{2}$$ (3)

where *i* is the current at a fixed potential *V*, and *v* is the scan rate in the CV tests, whereas *k*_1_ *v* and *k*_2_ *v* ^1/2^ indicate the surface capacitive and diffusion-controlled process, respectively. The *k*_1_ and *k*_2_ can be facilely obtained via plotting *i*(*V*)/*v* ^1/2^ (details are shown in Supplementary Material S1). The corresponding results are presented in Figures 5G–I. The capacitive contribution ratio is 24.7% at 0.5 mV s^−1^ for CoS/C-LIBs, the ratio increases with the increase in scan rates and finally reaches 35.1% at 1.2 mV s^−1^. In the case of CoS/C-SIBs and CoS/C-PIBs, the capacitive contribution ratio also increases with the increase of scan rates. The corresponding capacitive ratio in CoS/C-SIBs ascends from 53.5% at 0.2 mV s^−1^ to 65.8% at 1.2 mV s^−1^, whereas in CoS/C–PIBs, the capacitive contribution rises from 28.7% at 0.2 mV s^−1^ to 51.2% at 1.2 mV s^−1^. Obviously, the capacity percentage of capacitive contribution generally shows a high value in SIBs. This phenomenon may be due to the unique pore size distribution of ZIF-derived CoS/C, which is beneficial to the surface adsorption of Na^+^ and the insertion of Li^+^ and K^+^ to varying degrees. Besides, from the capacity comparison of LIBs and PIBs, the insertion of K^+^ is limited to the surface of electrodes owning to its larger ionic radius, so the capacity caused by incomplete conversion reaction is lower than that of LIBs (Hosaka et al., 2020).

## Conclusion

In this work, we successfully prepared ZIF-derived CoS/C electrodes and realized unprecedented performance for LIBs, SIBs, and PIBs. The CoS/C nanocomposite with hollow hexagon structure can be obtained through synthesis with ZIF-67 template. The large interlayer spacing in CoS/C is conducive to the migration of alkali metal ions, especially for Na^+^ and K^+^ with large ionic radius. The hollow structures provide a large number of active sites and thus improve the storage capability of alkali metal ions. Furthermore, the porous carbon framework improves the conductivity of electrodes and effectively buffers the volume expansion produced in electrochemical cycling. Therefore, for alkali ion batteries, the CoS/C electrodes exhibit superior electrochemical performance, including high specific capacity, long-cycle life, and fast rate ability. The electrochemical performance of designed CoS/C anode indicates that it has great application potential in advanced energy storage devices such as ion batteries or ion capacitors.

## Data Availability

The original contributions presented in the study are included in the article/Supplementary Material, further inquiries can be directed to the corresponding authors.
