# Supplementary material for: Hollow CoS/C Structures for High-Performance Li, Na, K Ion Batteries
Source: Front Chem. 2022 Mar 10;10:845742. doi: 10.3389/fchem.2022.845742 (PMC8960294; doi:10.3389/fchem.2022.845742)
Supplement: Supplementary file 1 [file DataSheet1.docx]

***Supplementary Material***


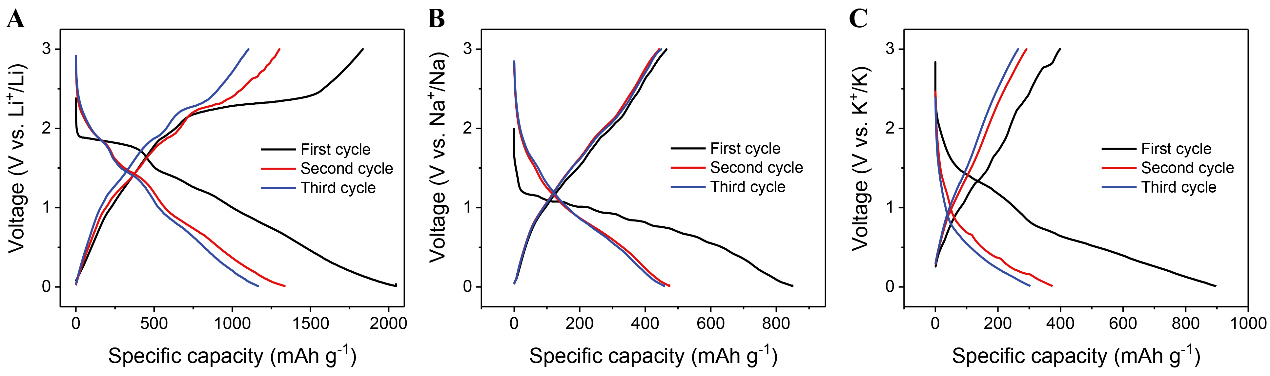


**Supplementary Figure S1.** Galvanostatic charge-discharge profiles of CoS/C electrodes of (A) LIBs, (B) SIBs and (C) PIBs.


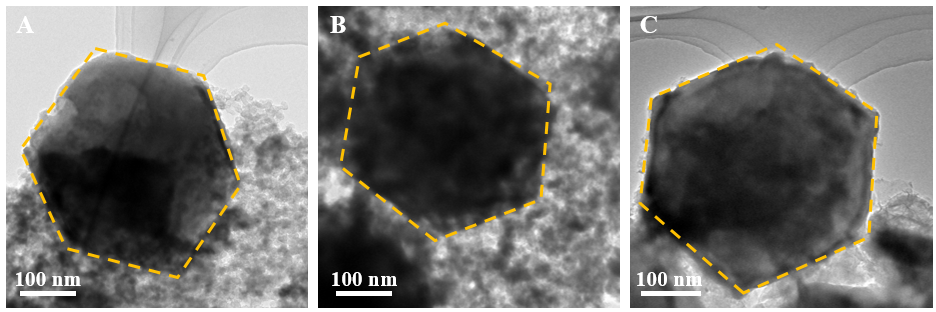


**Supplementary Figure S2.** TEM images of CoS/C electrodes of (A) LIBs, (B) SIBs and (C) PIBs after three cycles.


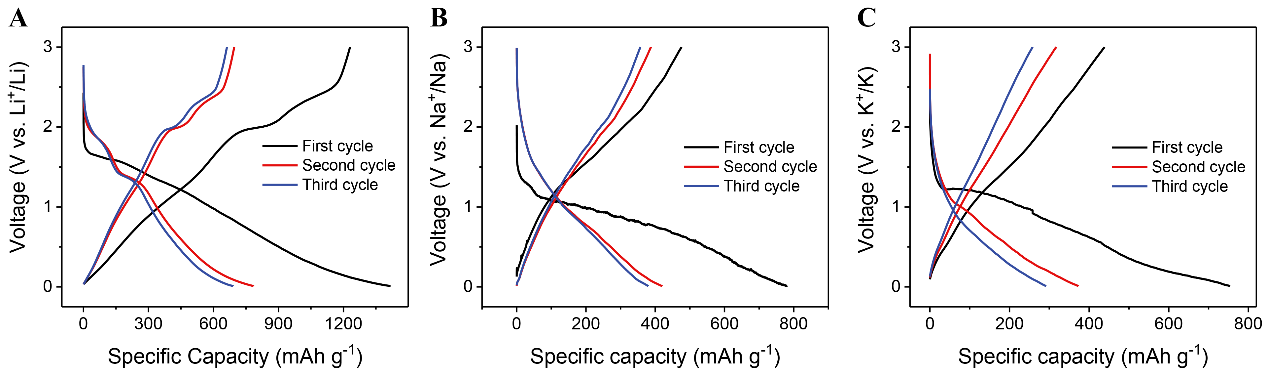


**Supplementary Figure S3.** Galvanostatic charge-discharge profiles of CoS electrodes of (A) LIBs, (B) SIBs and (C) PIBs.

**S1.** Details about the contribution of capacitive- and diffusion- controlled calculations

The total capacitance can be quantitatively distinguished at a given scan rate through the utilization of the following Equation (3). The k_1_ and k_2_ can be facilely obtained via plotting *i*(*v*)/*v*^1/2^. Under the specified voltage, the value of *k*_1_ can be obtained by linear fitting of *i*(*V*)/ *v*^1/2^ and *v*^1/2^ in Equation (3) by mathematical software such as “Origin”. Each fixed potential corresponds to a fitted *k*_1_ value and *k*_1_*ν* at the fixed potential is the contribution of the pseudocapacitance to the current. A number of fixed potentials are connected with *k*_1_*v* through a smooth curve, and the curve is nonlinearly fitted. Then, the area of the fitted closed curve was calculated by integral, and the area of the CV curve at a specific scanning rate can be also obtained by integral. The value obtained by dividing the area of the fitted curve by the area of the CV curve is the pseudocapacitance contribution rate at a specific scanning rate.
